# The complete chloroplast genome sequence of *Magnolia delavayi* (Magnoliaceae), a rare ornamental and medical tree endemic to China

**DOI:** 10.1080/23802359.2020.1717391

**Published:** 2020-01-27

**Authors:** Ao Liang, Wei Luo, Zhuoran Li, Yongkang Sima, Tao Xu

**Affiliations:** a College of Life Sciences, Yunnan University, Kunming, Yunnan, China;; b Yunnan Academy of Forestry, Key Laboratory for Conservation of Rare, Endangered &Endemic Forest Plants, Kunming, Yunnan, China

**Keywords:** Chloroplast genome, medical species, *Magnolia delavayi*, Magnoliaceae

## Abstract

*Magnolia delavayi* is a rare, famous ornamental and important medical tree endemic to China. Here, we assembled the complete chloroplast (cp) genome sequence of *M. delavayi*. Its length is 159,715 bp with four sub-regions: 87,906 bp of large single-copy region and 18,761 bp of small single-copy region are separated by two inverted repeats regions, each 26,524 bp. The genome contains 77 protein-coding genes, 6 rRNAs, and 29 tRNAs genes. Phylogenetic analysis of cp genome of *M. delavayi* with previously reported chloroplast genomes in *Magnolia* shows that *M. delavayi* is close to *M. odoratissima* with high bootstrap value.

*Magnolia delavayi* Franchet, an evergreen tree, endemic to China, Yunnan, Guizhou, Sichuan provinces (Xia et al. 2008) is a famous Chinese traditional medicine plant and is also receiving great concern for the anticancer activities of its leaf extracts sesquiterpenes (Xie et al. 2019). Apart from the medical value, it is also a rare well-known ornamental cultivated plant in the southwest of China (Figlar and Nooteboom 2004, Xia et al. 2008). In 2004, *Ma. delavayi* was listed as Vulnerable in the China Species Red List (Wang and Xie 2004), and in 2014, it was classified as ‘Least Concern’ in the IUCN Red List of Threatened Species due to the excessive anthropogenic collection and habitat destruction (Rivers and Wheeler 2014). However, *M. delavayi* is known as *Lirianthe delavayi* in China (Xia et al. 2008), only the pollination, biological characteristics of its cultivated plants (Gong et al. 1998), the number of ovules (Gong et al. 1999), and the artificial cultivation technology (Tang et al. 2014) have been studied. Here, the annotated cp genome sequence has been assembled and submitted to the GenBank with the accession number MN783014. The fresh leaves of *M. delavayi* were collected from a tree cultivated in Qiongzhu temple in Kunming city (25.07 N 102.62 E). The voucher specimen was deposited at the herbarium of the Institute of Botany, Chinese Academy of Sciences (PE00102017).

Total genomic DNA was extracted from the fresh leaves using Rapid Plant Genomic DNA Isolation Kit. The extracted DNA was sequenced using the Illumina Miseq platform (Illumina, San Diego, CA, USA). In total, 20.5 M of 150-bp raw reads were retrieved. In order to ensure the quality of information analysis, the original reads must be filtered to get clean reads using Trimmomatic (Bolger et al. 2014). Sequencing data were assembled with SPAdes (Bankevich et al. 2012) and GapFiller (Boetzer and Pirovano 2012) was used to supplement the GAP of the contig obtained by stitching. The genome was automatically annotated using Prokka (Seemann 2014). OGDRAW v1.3.1 (Greiner et al. 2019) was used to generate a physical map of the cp genome.

The length of the complete chloroplast (cp) genome sequence of *M. delavayi* is 159,715 bp with four sub-regions: 87,906 bp of LSC (large single-copy) region and 18,761 bp of SSC (small single-copy) region are separated by two IR (inverted repeats) regions, each 26,524 bp, akin to other taxa in the family of Magnoliaceae (Ling and Zhang 2019). The cp genome contained 112 genes, including 77 protein-coding genes, 29 tRNA genes, and 6 rRNA genes. Inverted repeat regions contain 15 genes (seven protein-coding genes, three rRNAs, and five tRNAs). The complete chloroplast genome of 10 reported Magnolia species and one Liriodendron species as an outgroup were downloaded from NCBI GenBank for phylogenetic analysis. The combined datasets of 12 species were aligned by Kalign (Madeira et al. 2019). A maximum-likelihood (ML) tree was constructed in MEGA v7.0 (Kumar et al. 2016) with 1000 bootstrap replications. The phylogenetic tree reveals that *M. delavayi* is most closely related to *M. odoratissima* with strong bootstrap support (Figure 1). The complete chloroplast cp genome resource of *M. delavayi* will be valuable in genetics conservation, pharmaceutical developments, and breeding in *Magnolia* species.

**Figure 1.**
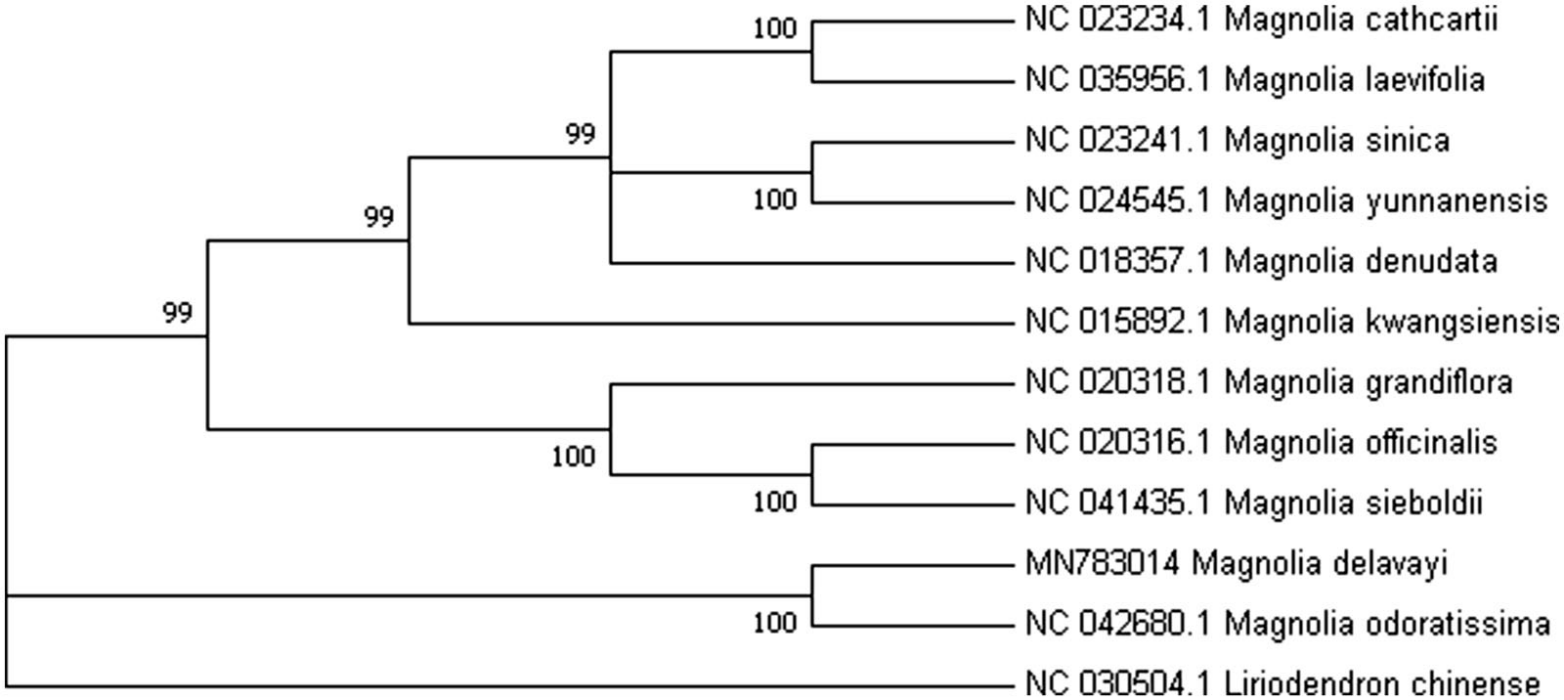
Phylogenetic tree using maximum-likelihood (ML) method based on the complete chloroplast genome of 12 species. Numbers above the node indicate bootstrap value.
